# P138: Soap and handrub consumption survey in Fann Teaching Hospital in Dakar

**DOI:** 10.1186/2047-2994-2-S1-P138

**Published:** 2013-06-20

**Authors:** NM Dia, D Faye, BA Niang, M Seydi

**Affiliations:** 1 Infectious Diseases Department, Fann Teaching Hospital, Fann, Senegal; 2 Central Pharmacy, Fann Teaching Hospital, Fann, Senegal

## Introduction

The prevention of Health care-associated infections (HAI) remains a stake in Public health. The hand is the main mode of transmission of microorganisms. So, hand hygiene is considered to be the primary measure necessary for reducing HAI.

## Objectives

The objective of our work is to measure the consumption of Alcohol-bases-handrub (ABHR) and soap intended for hand hygiene.

## Methods

A prospective investigation before the intervention was realized over a period of six months, of October 1st, 2011 to March 31 2012, with nine clinical departments of a hospital with 347 beds, where a manufacturing unit of ABHR in its WHO formulation was set up under the aegis of the APPS WHO program. The index form finalized by WHO was used.

## Results

In this public tertiary care hospital which employs 360 permanent nursing staff, the monthly average attendance is 2620 patients and number of admissions of 800 a month. During the study period, 77 % of the investigated departments used the ABHR among which 57 % in the form of solutions, 28 % in the form of gels and 15 % in the form of gels and solutions. The supply in ABHR was made in 58 % in the manufacturing unit of the central pharmacy. The monthly average ABHR consumption of the structure was 14.78 liters for a expected quantity of 1635 liters; that of liquids soaps 192 liters; that of soap bars18.6 liters. The average composite indicator of consumption of ABHR was 7.82 % with a minimum monthly use in the Emergency department and a maximum of 30.22 % in the oral department. The FANN teaching hospital was consequently classified E according to the achievement of the personalized objective, that is an establishment under 10 % of ICSHA.

## Conclusion

In spite of the existence of a manufacturing unit of ABHR, this consumption is still very low. The implementation of the WHO multimodal hand hygiene improvement in particular raising awareness and training, will help in a better compliance and use.

## Disclosure of interest

None declared

